# Preventing mpox at its source: Using food safety and One Health strategies to address bushmeat practices

**DOI:** 10.1186/s44263-024-00100-2

**Published:** 2024-10-09

**Authors:** Steven Lâm, Sherril Phyllis Masudi, Ha Thi Thanh Nguyen, Delia Grace

**Affiliations:** 1 International Livestock Research Institute, Naivasha Road, Nairobi P.O. BOX 30709-00100 Kenya; 2 International Livestock Research Institute, Hanoi, Vietnam; 3 Wildlife Ecology and Conservation Group, Wageningen University and Research, Wageningen, Netherlands; 4 Department of Medical Biochemistry and Microbiology, Uppsala University, Uppsala, Sweden; 5 Natural Resources Institute, University of Greenwich, Greenwich, UK

A key step in preventing mpox at its source is to address the handling and consumption of bushmeat from potential animal reservoirs. Changes to these practices must consider social, economic, and environmental factors, making a collaborative One Health approach essential. We propose a pilot intervention as a way forward.

## Background

Alarmingly, the World Health Organization (WHO) declared mpox (formerly monkeypox) a public health emergency of international concern in August 2024 [1]. This decision was prompted by a surge in cases in the Democratic Republic of Congo and other parts of Africa, the potential for further spread within and beyond the continent, and the emergence of a new strain of the virus. WHO had previously declared mpox an emergency in July 2022, remaining until May 2023 when the situation had been considered under control.

The strategies recommended by WHO and other major institutions call for immediate financial contributions—up to 4 billion USD—to support pandemic prevention and control efforts, which include, among others, surveillance, risk communication, and immunization [2, 3]. While these measures—which can be viewed as downstream activities—are vital for preventing human-to-human transmission of mpox, it is equally important to address upstream activities.

Preventing outbreaks is typically more cost-effective than managing them. Therefore, attention must be given to the root causes of disease emergence, particularly where mpox is transmitted from animals to humans. Human interaction with bushmeat—any tissue from wild animals used as food—is a major factor in the spread of mpox in regions where animals carry the virus. A One Health approach offers a promising, cost-effective framework, promoting collaboration across sectors to address the connections between humans, bushmeat, and the environment [2].

Over the last 10 years (2014–2024), there have been 16 mpox spillover events linked to bushmeat, which is more than in previous decades (see Additional file 1 for an overview). Most of these cases occurred in Africa, except for four cases involving travelers from Nigeria to the UK (*n* = 2), Singapore (*n* = 1), and Israel (*n* = 1). Where gender and age data were available, children made up the majority of those affected (48%), followed by men (34%) and women (18%). Rats were the most reported type of bushmeat involved, followed by squirrels and monkeys. Most spillover events did not result in large-scale outbreaks, and both handling and consumption were commonly linked to these cases.

### Applying lessons from food safety efforts in informal markets

Reducing risky interactions with bushmeat could help prevent mpox and other foodborne diseases early on. Bushmeat is often sold alongside other fresh foods in informal or “wet” markets, meaning traditional, non-modern food sale points. Since these markets and bushmeat activities typically operate with minimal regulation and inconsistent hygiene, applying advances in practices and policies from food safety research in informal markets could help mitigate the risks posed by bushmeat.

Based on two decades of research by the CGIAR—a global research partnership dedicated to transforming food, land, and water systems in a climate crisis—and partners in these hard-to-reach markets [4], the “three-legged stool” approach was developed. This approach posits that food safety can be improved if, and only if, three areas are addressed: [1] building capacity of value chain actors through training and simple technologies, [2] motivating behavior change through incentives and nudges, and [3] improving policies and regulations. Each component is essential for adopting safer practices. We expand below on how this approach could apply to bushmeat.

### Capacity-building

Food safety efforts in informal markets have traditionally focused on training local communities to encourage the adoption of safer practices. To design effective programs, frameworks like adult learning theory, social behavior change communication, and behavioral economics can provide valuable guidance but are little used [5].

Importantly, understanding how people perceive disease risk and what influences these perceptions is key to creating effective strategies. In communities where people already recognize the risks associated with bushmeat, health messages could focus on practical, protective steps. In places where skepticism exists, sharing evidence of health risks may be more successful. Since exposure to mpox can vary based on factors like gender, occupation, and other social identifiers, prevention strategies should be tailored accordingly [6].

Health messages need to be tailored to the local context. In the Democratic Republic of Congo, for example, there was distrust of formal institutions and a rejection of government health messages linking Ebola to bushmeat [7]. This suggests that risk reduction strategies from these sources may face challenges in gaining acceptance. Reaching at-risk populations may be more effective through a mix of formal and informal channels and working with trusted community leaders and healthcare staff.

Messages should also consider the significance of bushmeat to those involved [8]. Instead of pushing an anti-hunting agenda, a more helpful approach could involve providing ways to reduce the risk of disease transmission without completely discouraging hunting and consumption. While this approach may not eliminate all risks, it is likely to be more effective than a campaign that fails to resonate with the community.

In some settings, even when people are aware of the risks and know how to protect themselves, they may not adopt safer practices. While having the right knowledge is important for encouraging change, there also need to be incentives for people to act on what they know.

### Motivation and incentives

In low-resource settings, governments have frequently relied on bans and enforcement measures, including fines and inspections, as “incentives” for change. For example, the Nigerian government banned the sale of bushmeat as a precaution to stop the spread of mpox in June 2022. However, these approaches can have unintended consequences, such as driving bushmeat practices underground and worsening hygiene conditions [9].

A potentially more effective incentive is to focus on economic, social, or moral gains. Economic incentives, for example, might include describing the potential financial gains from attracting a larger customer base due to the credibility of the safer product. Social incentives could involve earning the trust and positive reputation of community members. Moral incentives could stem from the pride in ensuring that bushmeat is handled and sold in a way that reduces health risks. Involving key stakeholders—such as communities, government institutions, and national research centers—in the process helps ensure that risk prevention measures are locally validated and practical.

Although food safety is a large concern for consumers worldwide, it often takes a back seat to affordability among budget-constrained individuals. For these consumers, food safety is not a priority compared to cost. To ensure the sustainability and long-term effectiveness of food safety interventions, support from authorities is essential. Governments need to increase consumer awareness about food safety issues and make the choice of safer food options more accessible.

### Enabling policies and regulations

Local and national authorities can foster a supportive environment by investing in essential infrastructure, capacity building, monitoring, and surveillance activities. In some low-resource settings, food safety laws are either nonexistent or not applicable to informal contexts. Implementing tailored policies and regulations for bushmeat, particularly for high-risk settings, can improve hygiene efforts. Establishing recognition programs for businesses that achieve notable improvements in food safety can inspire others to follow their lead. Additionally, promoting alternative protein sources by providing access to affordable, nutritious food options and supporting sustainable agricultural practices can help to reduce bushmeat reliance.

### The role of One Health

Bushmeat is a key resource for many rural communities. In Africa, the annual harvest, estimated between 1 and 5 million metric tonnes, is substantial compared to the continent’s livestock production of about 14 million metric tonnes per year [10]. Climate change may further increase reliance on bushmeat as a food source. Any proposed changes to bushmeat practices must consider these social, economic, and environmental factors. The One Health approach promotes collaboration among public health authorities, veterinarians, wildlife experts, environmental scientists, and community leaders, enabling balanced measures to reduce transmission at the animal-human-environmental interface [2].

As countries plan their responses to mpox, three key considerations should be kept in mind. First, it is important to recognize that bushmeat is a crucial part of many communities' lives and contributes to their health and well-being. Second, responses should be developed with input from local communities to minimize any impacts of voluntary behavior changes, which will increase the chances of successful adoption; the One Health approach can help with this by bringing different actors together. Lastly, high-income countries should lead by not only sharing knowledge but also boosting funding for global health initiatives, as this investment can substantially reduce the risk of future outbreaks.

### A proposed pilot intervention

We propose a pilot project using the “three-legged stool” approach and leveraging One Health collaboration. First, identify a high-risk area for spillover and one amenable to interventions through epidemiological risk-targeting and socio-cultural characterization. Next, develop a proof-of-concept that includes (a) intensive sensitization and training for communities and local authorities; (b) providing subsidies to high bushmeat users for purchasing livestock or other alternative products, with subsidies linked to measurable improvements in biodiversity and reductions in hunting; and (c) engaging national decision-makers to secure buy-in. If successful, we suggest further refinement to maximize benefits, minimize costs, and extend the approach.

## Supplementary information

Supplementary Material 1. Table S1. Summary of spillover events of mpox (up to August 2024).

## Data Availability

No datasets were generated or analysed during the current study.

## Abbreviations

WHO: World Health Organization
