# Supplementary material for: Preventing mpox at its source: Using food safety and One Health strategies to address bushmeat practices
Source: BMC Glob Public Health. 2024 Oct 9;2:69. doi: 10.1186/s44263-024-00100-2 (PMC11622920; doi:10.1186/s44263-024-00100-2)
Supplement: Supplementary file 1 — Supplementary Material 1. Table S1. Summary of spillover events of mpox (up to August 2024). [file 44263_2024_100_MOESM1_ESM.docx]

**Additional file 1**

**Table S1.** Summary of spillover events of mpox (up to August 2024).

| Country | Year | Outbreak size | Case Detail | Exposure pathway | | Reference |
| --- | --- | --- | --- | --- | --- | --- |
|  |  |  |  | Handling | Consumption |  |
| Nigeria | 2022 | 1 | The case (male adult) was the first mpox case recorded in Adamawa, State Nigeria; the authors mentioned being unable to confidently link this case with any exposure due to an “inconsistent” history of contact with animals or visit to the bush provided by the patient. | (x) |  | (Pembi et al. 2022)* |
| United Kingdom (contracted in Nigeria) | 2022 | 1 | The case (male adult) traveled to Delta State in Nigeria. While the source of infection is unknown, the authors mentioned risks from contact with sick animals and eating or handling wild game. | (x) | (x) | (Hraib et al. 2022)* |
| Cameroon | 2022 | 1 | The case (boy) had caught a forest rat one week previously and prepared it before he and his family ate it. |  | x | (Jarman et al. 2022)* |
| Nigeria | 2022 | 33 (but not all linked to bushmeat) | 33 cases (12 men, 3 women, 18 children); exposure and possible sources of infection include the presence of rodents in and around households and contact with someone with rashes. | x |  | (Stephen et al. 2022)* |
| Singapore (contracted in Nigeria) | 2019 | 1 | The case (male adult) reported the consumed barbecued bushmeat that might have been contaminated. |  | x | (Yong et al. 2020)** |
| Israel (contracted in Nigeria) | 2018 | 1 | The case (male adult) reported during his last trip to Nigeria he had disposed of two rodent carcasses at his residence. | x |  | (Erez et al. 2019)* |
| United Kingdom (contracted in Nigeria) | 2018 | 2 (but not all linked to bushmeat) | The case (male adult) reported contact with a person with a mpox-like rash at a large family event as well as the consumption of bushmeat. |  | x | (Vaughan et al. 2018)**, *** |
| Central African Republic | 2018 | 6 | The index case (adult woman) reported butchering 3 small mammals, including African civit, pouched rat, and African rope squirrel. | x |  | (Besombes et al. 2019)*** |
| Nigeria | 2018 | 122 (but not all linked to bushmeat) | Among 122 confirmed cases, two cases (sex and age not specified) reported contact with an unspecified wild animal as well as bushmeat consumption. | x | x | (Yinka-Ogunleye et al. 2019)** |
| Democratic Republic of Congo | 2017 | 22 (but not all linked to bushmeat) | Among the confirmed 22 cases, four cases (2 girls, 2 boys) were from the same family, and reported being in contact with one another and having consumed bush meat. The index patient was a 14-year-old girl. Her family described consuming bushmeat, including small rodents, and the index patient reportedly handled and prepared the rodents before the onset of illness. | x | x | (Doshi et al. 2019)**, *** |
| Liberia | 2017 | 2 | Two cases of mpox, one confirmed and one suspected. The confirmed case was an 8-year-old boy. His mother is the suspect/primary case. There was no clear information on whether she had consumed or interacted with bush meat; however, her husband is a hunter. | (x) | (x) | (Larway et al. 2021)** |
| Nigeria | 2017 | 61 (but not all linked to bushmeat) | A total of 172 suspected and 61 confirmed cases were reported from 14 states in Nigeria. The authors state that mpox may be linked to a lack of food safety hygiene from bushmeat consumption. |  | X | (Okareh and Morakinyo 2018)** |
| Sierra Leone | 2017 | 1 | The case (male adult) had been hunting and eating squirrels for about 10 days before falling ill. |  | (x) | (Ye et al. 2019)**, *** |
| Central African Republic | 2016 | 26 (but not all linked to bushmeat) | The case (male adult) was a hunter and farmer, just like many other inhabitants of the village. He had consumed meat that came from the *Xerus erythropus* species of squirrels, found dead in the forest. |  | (x) | (Kalthan et al. 2018)**, *** |
| Central African Republic | 2016 | 10 | Ten people (3 children, 3 men, 4 women) were infected. The index case was a 9-year-old boy who fell sick after killing and cutting up a rodent known locally as “cibissi” and identified as *Thryonomis*. | x |  | (Nakoune et al. 2017)*** |
| Sierra Leone | 2014 | 1 | The case (boy) had had any history of contact with animals. However, both the mother and father of the boy stated that they regularly prepare and consume meat from wild animals. The mother and father also confirmed that small rodents were sometimes present in the family house. | x | x | (Reynolds et al. 2019)** |
| Democratic Republic of Congo | 2011-2012 | 3 | Of the three cases (2 men, 1 woman), case 1 noted contact with bushmeat before the onset of the disease; case 2 handled monkeys killed by local hunters and stored and ate monkey meat for his trip. | x | x | (McCollum et al. 2015)**, *** |
| Central African Republic | 2010 | 2 | Two cases (2 children) with lesions developed after hunting and eating a wild rodent. |  | x | (Berthet et al. 2011)**, *** |
| Democratic Republic of the Congo | 2001 | 16 (but not all linked to bushmeat) | The source of infection identified could be a monkey found dead in the forest that was handled and eaten by concerned family members. | x | x | (Meyer et al. 2002)**, *** |
| Democratic Republic of the Congo | 1996-7 | 511 (but not all linked to bushmeat) | Multiple index cases with shared exposure – all had consumed monkey, gazelle, pig, and rat. Serological testing confirmed the presence of antibodies within local squirrel, rat, shrew, and (domestic) pig species. Eating porcupines was the only exposure found to be statistically significant in index cases. | x | x | (Aplogan et al. 1998; Hutin et al. 2001)*** |
| Central African Republic | 1984 | 6 | In a Pygmy community, 6 cases (1 man, 5 children) were observed in two families: five children and a young woman. The head of the family had hunted a monkey with pustules on its body, and an antelope with the same type of lesions, whose flesh had been shared between the different families of the clan. | x | x | (Chastel and Charmot 2004)** |
| Democratic Republic of Congo | 1983 | 5 | Five cases (all children), two of which allegedly ate a monkey and a Gambian rat, and their respective families. |  | x | (Jezek et al. 1986)** |
| Zaire | 1972 - 1985 | 107 (but not all linked to bushmeat) | Transmission through food is mentioned as the main source of infection. The authors state that one of the factors of infection is “the method of food preparation”. In the Bumba area, 107 human cases of mpox were recorded from 1972 to 1985, while no cases were reported in the entire western region (Bas-Zaïre). | x | x | (Khodakevich, Jezek, and Messinger 1988)** |
| Liberia | 1970 | 1 | Four index cases, all children aged 4-9 years old, lived in the tropical rain forest where hunting of monkeys and duiker common. Three of the children were observed to play with the internal organs of recently killed monkeys. |  | x | (Foster et al. 1972)**, *** |
| Sierra Leone | 1970 | 1 | No evidence of consumption, mention of one case of mpox (male, 24 years old) who occasionally consumed monkeys. |  | x | (Foster et al. 1972)**, *** |
| Democratic Republic of the Congo | 1970-79 | 47 (but not all linked to bushmeat) | All index cases occurred within tropical rainforest areas where hunting of wild animals for food is common. The source of infection still unknown. Infection in local monkey, rodent and bird populations confirmed. | (x) | (x) | (Breman, Ruti, and Steniowski 1980; Heymann, Szczeniowski, and Esteves 1998)*** |
| Democratic Republic of the Congo | 1970 | 1 | The index patient was a 9-month-old child. Monkeys are commonly eaten by the patient’s family, with close contact with children/adults as the meat is caught/prepared. Infection in the local monkey population confirmed. | (x) | (x) | (Ladnyj, Ziegler, and Kima 1972; Marennikova et al. 1972)*** |

* From the current study.
** From the review of Chaix et al. (2022).
*** From the review of Milbank and Vira (2022).
Note: shaded cells represent cases before 2014 which were captured here for context.
Note: x represents the actual exposure route (explicitly stated) whereas (x) represents the likely exposure route (based on context provided).

**References**

Aplogan, A. et al. 1998. “Human Monkeypox - Kasai Oriental, Democratic Republic of Congo, February 1996-October 1997.” *JAMA*.

Berthet, Nicolas et al. 2011. “Maculopapular Lesions in the Central African Republic.” *The Lancet*.

Besombes, Camille et al. 2019. “Intrafamily Transmission of Monkeypox Virus, Central African Republic, 2018.” *Emerging Infectious Diseases*.

Breman, J. G., K. Ruti, and M. V. Steniowski. 1980. “Human Monkeypox, 1970-79.” *Bulletin of the World Health Organization*.

Chaix, Estelle et al. 2022. “Risk of Monkeypox Virus (MPXV) Transmission through the Handling and Consumption of Food.” *Microbial Risk Analysis*.

Chastel, C, and G Charmot. 2004. “[Bacterial and Viral Epidemics of Zoonotic Origin; the Role of Hunting and Cutting up Wild Animals].” *Bulletin de la Societe de pathologie exotique (1990)*.

Colquhoun, Heather L. et al. 2014. “Scoping Reviews: Time for Clarity in Definition, Methods, and Reporting.” *Journal of Clinical Epidemiology* 67(12): 1291–94.

Doshi, Reena H. et al. 2019. “Epidemiologic and Ecologic Investigations of Monkeypox, Likouala Department, Republic of the Congo, 2017.” *Emerging Infectious Diseases*.

Erez, Noam et al. 2019. “Diagnosis of Imported Monkeypox, Israel, 2018.” *Emerging Infectious Diseases*.

Foster, S. O. et al. 1972. “Human Monkeypox.” *Bulletin of the World Health Organization*.

Heymann, David L., Mark Szczeniowski, and Karin Esteves. 1998. “Re-Emergence of Monkeypox in Africa: A Review of the Past Six Years.” *British Medical Bulletin*.

Hraib, Munawar et al. 2022. “The Outbreak of Monkeypox 2022: An Overview.” *Annals of Medicine and Surgery*.

Hutin, Y. J. et al. 2001. “Outbreak of Human Monkeypox, Democratic Republic of Congo, 1996 to 1997.” *Emerging infectious diseases*.

Jarman, E. L., M. Alain, N. Conroy, and L. A. Omam. 2022. “A Case Report of Monkeypox as a Result of Conflict in the Context of a Measles Campaign.” *Public Health in Practice*.

Jezek, Z. et al. 1986. “Four Generations of Probable Person-to-Person Transmission of Human Monkeypox.” *American Journal of Epidemiology*.

Kalthan, E. et al. 2018. “Investigation of an Outbreak of Monkeypox in an Area Occupied by Armed Groups, Central African Republic.” *Medecine et Maladies Infectieuses*.

Khodakevich, L., Z. Jezek, and D. Messinger. 1988. “Monkeypox Virus: Ecology and Public Health Significance.” *Bulletin of the World Health Organization*.

Ladnyj, I. D., P. Ziegler, and E. Kima. 1972. “A Human Infection Caused by Monkeypox Virus in Basankusu Territory, Democratic Republic of the Congo.” *Bulletin of the World Health Organization*.

Larway, Lawrence Zegbain et al. 2021. “An Outbreak of Monkeypox in Doedain District, Rivercess County, Liberia, June, 2017.” *Journal of Interventional Epidemiology and Public Health*.

Marennikova, S. S., E. M. Seluhina, N. N. Mal’ceva, and I. D. Ladnyj. 1972. “Poxviruses Isolated from Clinically Ill and Asymptomatically Infected Monkeys and a Chimpanzee.” *Bulletin of the World Health Organization*.

McCollum, Andrea M. et al. 2015. “Case Report: Human Monkeypox in the Kivus, a Conflict Region of the Democratic Republic of the Congo.” *American Journal of Tropical Medicine and Hygiene*.

Meyer, Hermann et al. 2002. “Outbreaks of Disease Suspected of Being Due to Human Monkeypox Virus Infection in the Democratic Republic of Congo in 2001.” *Journal of Clinical Microbiology*.

Milbank, Charlotte, and Bhaskar Vira. 2022. “Wildmeat Consumption and Zoonotic Spillover: Contextualising Disease Emergence and Policy Responses.” *The Lancet Planetary Health*.

Nakoune, Emmanuel et al. 2017. “A Nosocomial Outbreak of Human Monkeypox in the Central African Republic.” *Open Forum Infectious Diseases*.

Okareh, O.T., and O.M. Morakinyo. 2018. “Monkeypox in Nigeria: A Case Report of Re-Emerged Disease Outbreak.” *Journal of Microbiology & Experimentation*.

Pembi, Emmanuel et al. 2022. “First Confirmed Case of Monkeypox in Adamawa State, Nigeria: A Clinico-Epidemiological Case Report.” *Pan African Medical Journal*.

Reynolds, Mary G. et al. 2019. “Human Monkeypox in Sierra Leone after 44-Year Absence of Reported Cases.” *Emerging Infectious Diseases*.

Stephen, Roland et al. 2022. “The Epidemiological Trend of Monkeypox and Monkeypox-Varicella Zoster Viruses Co-Infection in North-Eastern Nigeria.” *Frontiers in Public Health*.

Vaughan, Aisling et al. 2018. “Two Cases of Monkeypox Imported to the United Kingdom, September 2018.” *Eurosurveillance*.

Ye, Fei et al. 2019. “Molecular Evidence of Human Monkeypox Virus Infection, Sierra Leone.” *Emerging Infectious Diseases*.

Yinka-Ogunleye, Adesola et al. 2019. “Outbreak of Human Monkeypox in Nigeria in 2017–18: A Clinical and Epidemiological Report.” *The Lancet Infectious Diseases*.

Yong, Sarah Ee Fang et al. 2020. “Imported Monkeypox, Singapore.” *Emerging Infectious Diseases*.
